# Expanding the molecular epidemiology of melioidosis in North Central Vietnam

**DOI:** 10.1371/journal.pntd.0013945

**Published:** 2026-02-09

**Authors:** Michael H. Norris, Thi Hai Au La, Morgan C. Metrailer, Ha Viet Nguyen, Quyen Thi Le Tran, Treenate Jiranantasak, Tan Minh Luong, Andrew P. Bluhm, Bich Ngoc Do, Thi Thu Ha Hoang, Minh Hoa Luong, Thanh Hai Pham, Linh Nguyen Hai Bui, Hang Thi Thu Nguyen, Huyen Thi Pham, Trung Thanh Trinh, Jason K. Blackburn

**Affiliations:** 1 Spatial Epidemiology & Ecology Research Laboratory, Department of Geography, University of Florida, Gainesville, Florida, United States of America; 2 Emerging Pathogens Institute, University of Florida, Gainesville, Florida, United States of America; 3 Pathogen Analysis and Translational Health Group, School of Life Sciences, University of Hawaiʻi at Mānoa, Honolulu, Hawaiʻi, United States of America; 4 VNU-Institute of Microbiology and Biotechnology, Vietnam National University, Hanoi, Vietnam; 5 Department of Environmental Health, Faculty of Environmental and Occupational Health, Hanoi University of Public Health, Hanoi, Vietnam; 6 National Institute of Hygiene and Epidemiology, Hanoi, Vietnam; 7 Vietnam Institute of Animal and Veterinary Science, Hanoi, Vietnam; 8 Ha Tinh General Hospital, Ha Tinh, Vietnam; Mahidol Univ, Fac Trop Med, THAILAND

## Abstract

Cases of melioidosis have been recorded for many years in Vietnam though it is still not a nationally reportable disease in Vietnam. More research is needed to understand the disease ecology and public health impacts of melioidosis in the country. To this aim, broadening the knowledge base of strains and epidemiology of infections in relation to genotypes present in the soil reservoir can tell us about the propensity of *Burkholderia pseudomallei* genotypes to transmit from soil to humans. Thirty-five clinical *B. pseudomallei* isolates, ten from soil, one from swine, and one from a bear were collected by the Institute of Microbiology and Biotechnology, Vietnam National University and sequenced at the National Institute of Hygiene and Epidemiology in Hanoi. The clinical strains were isolated from melioidosis patients from Ha Tinh in each month of 2020 (except July). There were 15 STs identified and four of the clinical isolates were new sequence types (ST) as determined by traditional seven marker multi-locus sequence typing (MLST) analysis. Twenty of the thirty-five (57%) clinical strains isolated in this study were ST 41, with ST 41 isolates obtained throughout the year and across Ha Tinh province with core genome (cg) MLST identifying finer scale differences. ST 41 was recovered from one soil sample approximately 1 year after the clinical isolates. cgMLST analysis and whole genome SNP analysis revealed nucleotide differences among strains in Ha Tinh historically contextualizing them in Vietnam and globally. As melioidosis moves towards a reportable disease in Vietnam, molecular epidemiological methods can connect human, veterinary, and environmental genotypes of concern.

## Author summary

The disease melioidosis has been intensely studied in Thailand and Northern Australia while many other locations have faced a lack of investigation, including other countries in Southeast Asia. Among the lesser studied areas is Vietnam. The bacterium that causes melioidosis, *Burkholderia pseudomallei*, can cause severe infections and is a public health concern in wet tropical locations around the world. This study takes a detailed molecular epidemiological look at clinical, soil, and veterinary *B. pseudomallei* isolates from North Central Vietnam. The isolates were whole genome sequenced which revealed STs shared among soil, veterinary, and clinical *B. pseudomallei* strains. Clinical sequence types were heavily enriched for isolates that shared unique gene sets that could indicate geographically specific bacterial virulence mechanisms.

## Introduction

The disease melioidosis, caused by the bacterium *Burkholderia pseudomallei*, was first described in 1912 by A. Whitmore and C.S. Krishnaswami as a disease afflicting injectional morphine users in what was then Rangoon, Burma (now Yangon, Myanmar) [1]. It occurs across the globe in wet tropical environments with an evolving epidemiology [2–4]. The disease has been intensely studied in Thailand and Northern Australia [5–8] while many other locations have faced a lack of investigation, including other countries in Southeast Asia. Among the lesser studied areas is Vietnam, a country where melioidosis cases were associated with American soldiers returning from war thus earning the moniker “Vietnamese time bomb”. Melioidosis cases from travel to Vietnam have also been recorded [9]. Even with the preponderance of evidence that melioidosis exists in Vietnam, the true incidence of disease in humans, the spatial context of disease, and the genetics of strains present in the country remain to be fully understood.

*B. pseudomallei* genetic diversity has been studied with seven-locus multi-locus sequence typing (MLST) [10]. MLST analysis allows differentiation of the 2,220 sequence types (STs) currently identified. More recently MLST schemes targeting genes in the core genome of *B. pseudomallei* have been developed [11] where whole genome sequences are used for allelic typing at over four thousand genome loci. Currently there are more than 1,000 core genome sequence types (cgSTs) deposited in the public pubMLST database [12]. While vital epidemiological information can be derived from comparing cgSTs, availability of whole genome sequence allows single nucleotide polymorphism (SNP) analysis for highly detailed phylogenetic analysis. *Burkholderia pseudomallei* genetic variation has been intensely studied with data heavily skewed to Thailand and Northern Australia. The total number of *B. pseudomallei* isolates deposited in the seven-marker MLST sequence typing PubMLST database total 7,741. Of those isolates 1,163 and 3,903, from Thailand and Australia respectively, as of December 2025. The remaining ~34% of isolates are from the rest of the world, with 269 from Vietnam (inclusive of this study). PubMLST also hosts whole genomes. Of the 1,272 *B. pseudomallei* genomes deposited as PubMLST genomes, 874 are from Australia, 41 are from Thailand, and 55 are from Vietnam. In the NCBI sequence read archive there are 9,799 entries for *B. pseudomallei*. Australia has 1,760, Thailand 3,402 and Vietnam 139 (inclusive of this study). These numbers illustrate the overwhelming proportion of *B. pseudomallei* strain typing and sequencing done in Australia and Thailand, leaving much to be discovered in other countries where melioidosis is known to be endemic yet under studied, as in Vietnam [13]. A few molecular epidemiological studies of strains in Vietnam have been completed. Seven marker MLST analysis of four strains of *B. pseudomallei* collected in 1964 was an early, limited study on strain diversity in Vietnam [14]. More recent studies in Vietnam utilized genomic epidemiology to link a single human case to soil isolates in Nghe An province [11] and identified a contaminated bore-well as the source of *B. pseudomallei* that killed three children in Hanoi [15].

Geographic biases can affect epidemiology of melioidosis by limiting place-specific bacterial diversity and obscuring impact of patient ethnicity, while simultaneously ignoring differences in ecology and culture that play important roles in disease. The current work is the outcome of a multi-institutional project to increase the diagnosis of *B. pseudomallei* in Vietnam. By enhancing awareness and microbiological methods of diagnosis, new strains could be identified, isolated and whole genome sequenced. The more strains with whole genome sequence available the more details regarding strain diversity, transmission to humans and animals, and environmental persistence can be gained. To that end, increased capacity for diagnostic isolation of *B. pseudomallei* strains was implemented to improve the molecular epidemiology of melioidosis in the North Central Vietnamese province of Ha Tinh. The enhanced diagnostic techniques were employed in Ha Tinh Provincial General Hospital, and the increase in positively identified melioidosis samples throughout 2020 provided a series of clinical isolates for genome sequencing and molecular epidemiological analysis. Follow up environmental sampling and isolation of *B. pseudomallei* from soils in Ha Tinh provided genetic snapshots of bacterial diversity maintained in the soil. To complete our One-Health approach to characterizing melioidosis in Vietnam, two veterinary isolates, one from a pig and one from a bear, were sequenced. As the reporting of incidence increases in Vietnam, it is anticipated the disease will become nationally reportable allowing development and implementation of country wide public health policies around prevention, diagnostics, and treatment of melioidosis.

## Methods

### Ethics statement

Clinical cases were diagnosed during routine hospital procedures. The clinical data were retrieved based on bacterial cultures for this retrospective analysis. Medical records were deidentified and correlated with diagnostic microbiology findings. The retrospective studies of human medical records and microbiological isolates were approved by the University of Florida Institutional Review Board under protocol IRB# IRB202301112 and by the Institutional Review Board at the University of Medicine and Pharmacy, Vietnam National University under protocol IRB# IRB00013221. Animal sampling took place following Article 21 under the Vietnam National Law governing animal welfare. Swine sampling was performed by the Vietnam Institute of Animal and Veterinary Science under the guidance of the Ministry of Agriculture and Rural Development Circular 7.

### Study location, clinical records and diagnosis

**Fig 1:**
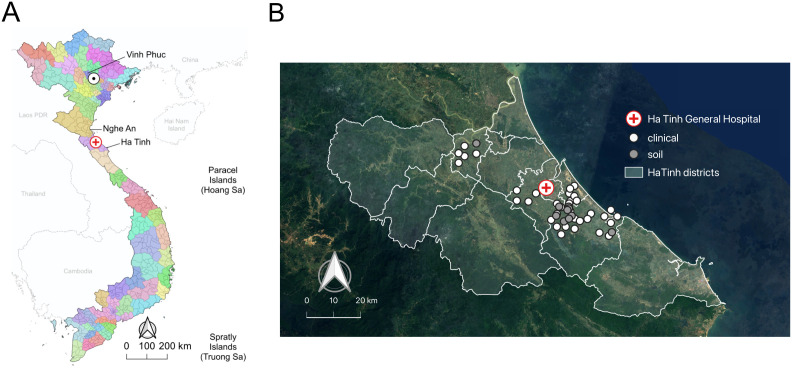
Locations of *B. pseudomallei* positive samples isolated during this study. **A)** Provinces are colored differently while the smaller subdivisions are districts. The provinces Vinh Phuc, Nghe An, and Ha Tinh are labeled. The white circle with the black dot indicates the location of Hanoi, Vietnam where IMBT and NIHE are located and the red plus sign indicates the location of Ha Tinh Provincial General Hospital in Ha Tinh province where the clinical and environmental strains were isolated. **B)** A map showing locations of clinical *B. pseudomallei* strains from Ha Tinh province. Clinical strains (white points) isolated in 2020 and environmental *B. pseudomallei* strains (gray points) from 2016 and 2022 in relation to Ha Tinh Provincial General Hospital (red cross) within Ha Tinh Province. White lines indicate districts within Ha Tinh. The names of provinces and district-level administrative divisions are those used before July 1^st^, 2025. Regional country basemaps (https://gadm.org/maps.html), country (https://purl.stanford.edu/dp747xv3917), province (https://purl.stanford.edu/mq929cm3505) and district (https://purl.stanford.edu/zq121fy4008) level base maps were used following the GADM license (https://gadm.org/license.html).

Ha Tinh Province is located in North Central Vietnam and Ha Tinh Provincial General Hospital is the provincial hospital that accepts patients primarily from the districts within Ha Tinh province (Fig 1A). A major component of the current project was increasing local knowledge and diagnostic capacity for melioidosis in hospitals of North Central Vietnam. This was accomplished by introducing more sensitive diagnostic techniques while educating laboratorians and clinicians through workshops and training. Increased isolation rates of *B. pseudomallei* from patients in Ha Tinh Provincial General Hospital allowed for molecular analysis of a series of clinical isolates across patient symptom presentations and syndromic outcomes at various locations in Ha Tinh Province (Fig 1B). The research collaboration allowed for rapid screening and molecular characterization of *B. pseudomallei* clinical isolates identified during routine patient care. Patients with high fevers and suspected melioidosis had their diagnostic blood samples processed for *B. pseudomallei* as previously described [16]. Briefly, 1 ml of patient whole blood was injected into blood bottles (BACT/ALERT FA Plus, Biomerieux, Marcy-I’Étoile, France) and incubated at 37°C until growth was observed. Bottles with growth were struck on to Ashdown’s selective agar and incubated at 37°C for up to 48 h [17]. Suspect colonies were Gram-stained and genomic DNA was extracted from colonies with rod-shaped Gram-negative bacteria. Positive identification of *B. pseudomallei* was confirmed using the TTS1-*orf2* quantitative polymerase chain reaction (qPCR) assay [18]. All province and district names and their administrative levels mentioned in this study refer to those in use before July 1st, 2025. This work was performed under University of Florida IRB# IRB202301112 and the University of Medicine and Pharmacy, Vietnam National University IRB# IRB00013221.

### Veterinary diagnosis

Two bacterial strains from animals were also sequenced during this analysis, including one from a pig in Nghe An, located near Ha Tinh in central Vietnam, and one from an Asiatic brown bear in Vinh Phuc, a mountainous province to the northwest of Hanoi [19]. Tracheal swabs from an underweight swine serologically positive by ELISA were plated on Ashdown’s agar to isolate colonies [20,21]. For the bear isolate, a pus swab from a lethargic bear was cultured at Central Hospital 74 in Vinh Phuc on routine media, including blood agar and MacConkey agar. After 2-days incubation at 37°C, growth was observed. In each case, isolated colonies with the *B. pseudomallei* phenotype were plucked and saved for qPCR confirmation and whole genome sequencing. Animal sampling took place following Article 21 under the Vietnam National Law governing animal welfare. Swine sampling was performed by the Vietnam Institute of Animal and Veterinary Science under the guidance of the Ministry of Agriculture and Rural Development Circular 7. The bear sample was collected by rural veterinarians following article 21 then transported to IMBT.

### Environmental sampling and bacterial isolation

Regional environmental samples from 2016 and 2022 (before and after the clinical cases) were from northern and central areas of Ha Tinh province where the cases were reported. In 2016, 80 samples at 16 randomly chosen sites were collected. In 2022, 50 soil samples at 10 sites in the Central Ha Tinh region proximate to areas with high case reports from 2020 and 50 soil samples at 10 sites where less melioidosis was reported in 2020 (Northeastern Ha Tinh) were collected. Soil samples were obtained from 30 cm below the surface using a JMC Soil Samplers soil sampling auger. At each sampling site, five soil samples were taken, with 5–10 m between each other. The steel auger used for collection was cleaned with bottled water and disinfected with 70% (v/v) ethanol after each sampling point. Approximately 200 g of soil sample was collected in plastic ziploc bags and transferred to the Institute of Microbiology and Biotechnology (IMBT), Vietnam National University, Hanoi. Samples were transported and stored at ambient temperature before processing within 1 week for *B. pseudomallei* isolation using the two-step enrichment method as previously described [22]. Briefly, 10 g of soil was transferred into 50-ml tube containing 20 ml TBSS-C50 broth. After vigorously vortexing, the tube was incubated statically at 40°C for 2 days. Then, 1 ml of the first culture supernatant was transferred to a new 50 ml tube containing 9 ml of erythritol medium (EM). The tube was incubated at 40°C for 4 days prior to plating on Ashdown’s agar. After incubation at 40°C for 4 days, suspected *B. pseudomallei* colonies were picked up and stored at -70°C in Luria-Bertani broth supplemented with 20% glycerol for further identification and genomic analysis.

### Mapping

GPS locations of clinical cases were recorded at diagnosis. The locations are confidential, and these data are not releasable under IRB regulations. For this study, patient locations were protected by dithering all points by 100 meters using the point displacement function in QGIS [23]. GPS coordinates for soil samples were also recorded, dithered, and mapped using QGIS. The names of provinces and district-level administrative divisions (provinces, districts) were those in place prior to July 1, 2025. The shapefiles used in this work are from the Global Administrative Areas 2015 (v2.8) dataset used under the CC BY 4.0 license and were accessed via the following urls on December 31, 2024: administrative level 0 (country) https://purl.stanford.edu/dp747xv3917, administrative level 1 (provinces) https://purl.stanford.edu/mq929cm3505, and administrative level 2 (districts) https://purl.stanford.edu/zq121fy4008. These data are from the GADM dataset which is freely available for publication (https://gadm.org/license.html). Satellite imagery was accessed on December 8, 2025 from the QGIS quick map services EOX::Maps Sentinel-2 cloudless map, which is sourced from the European Space Agency’s Sentinel-2 and publishable under CC BY 4.0.

### Bacterial isolation and qPCR confirmation

*Burkholderia pseudomallei* strains were isolated from patients visiting Ha Tinh Provincial General Hospital during 2020, from the two animals, and soils in 2016 and 2022. The genomic DNA was purified using the Promega Wizard Genomic DNA extraction kit and qPCR confirmed using the TTS1 qPCR assay [18,24] at IMBT at Vietnam National University.

### DNA sequencing

Libraries were prepared as previously described [25,26]. Briefly, sequencing libraries were generated using the Illumina Nextera XT DNA library preparation kit according to the manufacturer’s recommendations. DNA sequencing was performed at the National Institute of Hygiene and Epidemiology (NIHE) in Hanoi on the Illumina MiSeq system. The fastq files of the 47 strains sequenced have been deposited in the NCBI Sequence Read Archive under SRA BioProject PRJNA1180080.

### Bioinformatics and bacterial typing

Raw data were processed on the University of Hawai’i KOA High Performance Computing Cluster. Whole genome sequencing data were used to generate assembled contigs using the nextflow(nf)-core pipeline *bacass* [27,28]*.* This pipeline trims the raw reads based on quality using FastP [29] followed by read assembly with Unicycler [30] and annotation with Prokka [31]. Available fastq files from the NCBI sequence read archive (SRA) for 1468 strains were downloaded, assembled, and annotated by the same pipeline. Strains and metadata are indicated in S1 Table. The assembled genomes were typed by *in silico* standard MLST and cgMLST analysis using loci allele sequences and schema from pubMLST. Briefly, the *B. pseudomallei* K96243 genome was used to create a prodigal training file with chewBBACA [32]. All allele sequences for each locus were downloaded from pubMLST by API and allele calling was performed using the Coreugate nextflow pipeline [33]. The cgMLST profiles from the strains sequenced in this work were submitted to pubMLST and had cgSTs assigned for the comparison to strains in the database. Whole genome SNP analysis was used to identify molecular genetic patterns and to identify diagnostic targets. It was performed by analyzing the assembled whole genomes with PhaME, a pipeline developed at Los Alamos National Lab (LANL) [34]. Briefly, draft assemblies were aligned to the *B. pseudomallei* 1026b reference (GCA_000260515) using nucmer. SNP and gap locations are used to generate core genome alignments. Phylogenetic tree inference and ultrafast bootstrapping were performed with IQ-TREE by implementing the transversion with estimated base frequencies, invariant sites, gamma rate variation, model and incorporation of the proportion of invariable sites model (IQ-TREE option -m TEST found TVM + F + I + G4 was the best model as determined by AIC and BIC testing) [35]. The phylogenetic trees in Newick format were visualized in the standalone grapetree [36] module and with iTOL [37].

### Accessory gene analysis

Gene annotations strains were analyzed by the Roary pan-genome analysis pipeline [38] to identify genes or operons unique to the twenty-one ST 41 *B. pseudomallei* clinical isolates and one environmental isolate identified in this study. GFF3 files generated by Prokka from assemblies in this study were compared to forty-one closely related non-ST 41 strains downloaded from GenBank using default Roary parameters. The nucleotide sequences encoding for the proteins present only in ST 41 strains were extracted. Blastn of these sequences using the default settings with a 0.05 expected mismatch threshold was used to confirm absence or presence of these gene regions in other bacterial strains, including other *B. pseudomallei*. The gene region was considered as present if alignment coverage was > 90% and there was > 90% nucleotide identity.

## Results

*Burkholderia pseudomallei* was isolated from 35 melioidosis patients; 11 that had diabetes (31.4%) (Table 1). Eight of the patients died, eleven patients recovered, and the remaining were lost to follow up after transfer from the hospital. Initial diagnoses included septicemia, soft tissue abscesses, arthritis, pneumonia, pleural effusion, and a urinary tract infection. *Burkholderia pseudomallei* was isolated in blood from 26 of the patients, pus from seven, sputum from one, and pleural fluid from the last. Whole genome sequencing at NIHE in Hanoi allowed *in silico* sequence typing. Traditional seven-marker MLST identified nine STs total, including two new STs, among the clinical isolates. Surprisingly, 20 out of 35 strains (57%) isolated from the 35 patients across the year were ST 41. All other clinical STs were unique. Four of the clinical isolates were new STs. Strain 34HT was assigned ST 2215, 43HT was assigned ST 2216, and 10HT was assigned ST 2220. The fourth strain, 46HT matched ST 41 except the *lipA* sequence was not found. This region of the genome was not present, indicating a deletion encompassing the *lipA* locus. Whole genome SNP analysis and cgMLST placed this strain in the ST 41 cluster even though the *lipA* sequence was missing, bringing the total ST 41 strains to 21 out of 35 (60%). The earliest ST 41 strain, isolate 2002721102 from a 1983 human melioidosis case, was deposited in pubMLST by the US CDC, likely a travel related infection from Southeast Asia possibly even Vietnam [39]. During the preparation of this manuscript, the US CDC published a study revealing environmentally acquired US melioidosis cases occurring in 2024 in the state of Georgia [40]. The 2024 cases were linked spatially and genetically to the above mentioned 1983 melioidosis case. The closest related strains from this current work are 45HT from Cam Xuyen district and 48HT from Thach Ha district. Genetically they fall outside the main clades where the Georgia strains are located but indicate a potential common ancestor from Central Vietnam.

**Table 1:** Clinical isolate details.

Strain	Date of isolation	Isolation source	District	Diagnosis	Diabetes	Outcome	MLST ST
02HT	14.01.2020	Blood	Thạch Hà	septicemia	N	request discharge by severe infection^†^	41
05HT	25.02.2020	Pus	Cẩm Xuyên	soft tissue abscess	N	recovered	41
07HT	23.03.2020	Pus	Thạch Hà	soft tissue abscess	Y	recovered	221
10HT	09.04.2020	Pus	Cẩm Xuyên	septicemia	N	recovered	2220*
13HT	06.05.2020	Blood	Thạch Hà	septicemia/arthritis	N	transferred to another hospital	41
14HT	14.06.2020	Blood	Thạch Hà	pneumonia	N	request discharge by severe infection	41
15HT	22.06.2020	Sputum	Đức Thọ	multilobar pneumonia	Y	recovered	41
16HT	26.06.2020	Pus	Đức Thọ	soft tissue abscess	N	recovered	507
17HT	05.08.2020	Pus	Cẩm Xuyên	soft tissue abscess	N	transferred to another hospital	41
19HT	14.08.2020	Blood	Đức Thọ	pneumonia	Y	request discharge by severe infection	41
21HT	18.08.2020	Blood	Đức Thọ	UNKNOWN	N	transferred to another hospital	41
23HT	21.08.2020	Blood	Cẩm Xuyên	septicemia/pneumonia	Y	transferred to another hospital	41
26HT	30.08.2020	Blood	Cẩm Xuyên	septicemia/upper lobe pneumonia	N	recovered	691
27HT	22.09.2020	Blood	Thạch Hà	septicemia	N	transferred to another hospital	299
32HT	04.10.2020	Blood	Cẩm Xuyên	septicemia/multilobar pneumonia	N	transferred to another hospital	41
34HT	05.10.2020	Pus	Cẩm Xuyên	soft tissue abscess	N	recovered	2215*
40HT	25.10.2020	Blood	Cẩm Xuyên	septicemia/lower lobe pneumonia	N	request discharge by severe infection	41
42HT	30.10.2020	Blood	Thạch Hà	septicemia	N	recovered	1051
43HT	02.11.2020	Blood	Thạch Hà	pneumonia	N	transferred to another hospital	2216*
44HT	02.11.2020	Blood	Cẩm Xuyên	pneumonia	N	request discharge by severe infection	307
45HT	03.11.2020	Blood	Cẩm Xuyên	septicemia/pneumonia	N	transferred to another hospital	41
46HT	05.11.2020	Blood	Cẩm Xuyên	lower lobe pneumonia/UTI	N	transferred to another hospital	‡
47HT	06.11.2020	Blood	Cẩm Xuyên	pneumonia	N	transferred to another hospital	41
48HT	14.11.2020	Blood	Thạch Hà	septicemia	N	death	41
50HT	08.11.2020	Blood	Cẩm Xuyên	septicemia	Y	recovered	41
51HT	14.11.2020	Blood	Thạch Hà	septicemia/pneumonia	Y	death	41
53HT	17.11.2020	Blood	Cẩm Xuyên	septicemia	N	recovered	41
54HT	24.11.2020	Blood	Đức Thọ	septicemia	Y	transferred to another hospital	41
55HT	30.11.2020	Blood	Thạch Hà	septicemia/pleural effusion	N	transferred to another hospital	70
56HT	01.12.2020	Pus	Thạch Hà	septicemia/cheek abscess	Y	transferred to another hospital	41
57HT	30.11.2020	Blood	Thạch Hà	septicemia/pleural effusion	N	transferred to another hospital	392
58HT	04.12.2020	Blood	Cẩm Xuyên	septicemia/pneumonia	Y	request discharge by severe infection	1567
60HT	15.12.2020	Blood	Thạch Hà	septicemia	Y	recovered	654
61HT	22.12.2020	Blood	Cẩm Xuyên	septicemia/multilobar pneumonia	Y	transferred to another hospital	41
64HT	25.12.2020	Pleural fluid	Cẩm Xuyên	pleural effusion	N	UNKNOWN	197

**Clinical *B. pseudomallei* isolate table.** A table of relevant clinical, geographic, and genotypic information for *B. pseudomallei* isolated from patients during this study. Dates are in the day/month/year format. The 7 marker MLST types determined from *in silico* typing are listed in the column on the right. 20/35 clinical isolates were ST 41. Whole genome SNP and cgMLST typing placed this strain within the ST 41 clade bringing the presumptive ST 41 total to 21/35. ^†^; request discharge by severe infection- Families often request hospital discharge of a very sick individual whose recovery is unlikely. These individuals likely die at home though they are lost to follow-up. *; strains with new sequence types. ^‡^; strain 46HT lacked the *lipA* gene, precluding the *de facto* typing as ST 41.

Soil samples in Ha Tinh from 2016 and 2022, four years before and again two years after the clinical series, were processed for *B. pseudomallei* isolation to provide molecular environmental context to bacterial diversity in the area. *Burkholderia pseudomallei* strains were successfully isolated from 15 soil samples at eight randomly chosen sites from 2016 (S1 Fig and S2 Table). In 2022, 50 samples from 10 sites in an area coinciding with 2020 high human incidence were isolated in Central Ha Tinh. *Burkholderia pseudomallei* was successfully isolated from eight of ten of those sites (19 of 50 samples; S2 Table). Another 50 soil samples were isolated in Northeastern Ha Tinh where few human cases were reported. One sample of 50, at only 1 of 10 sites, were positive from this sampling area. In total, *B. pseudomallei* was isolated from 20 soil samples at 9 sites. A subset of 10 of 35 isolates were sequenced in this work, three from 2016 and seven from 2022. Two ST 56 strains were isolated in 2022. This sequence type has been found in the environment and in human infections across Southeast Asia, including Bangladesh, Thailand, Cambodia, Malaysia, and Vietnam. Among the *B. pseudomallei* soil isolates from the 2016 and 2022 sampling Ha Tinh events, two STs overlapped with the clinical STs. These two STs found in human patients and also the soil sampled during 2022 were STs 41 (strain SHT3.5) and 307 (strain SHT4.5G) (Table 2). Two of the soil isolates, both from 2016, had novel STs. Strain MT22.4 was assigned ST 2217 and strain MT27.4.1 was assigned ST 2218 by pubMLST. *Burkholderia pseudomallei* isolate VP74 G20.1 from the bear in Vinh Phuc had a novel ST assigned, ST 2219, while swine isolate TY01 from Nghe An was ST 55. Sequence type 55 was found in both the 2016 swine from Nghe An (strain TY01) and a 2022 Ha Tinh soil sample (strain SHT8.1). Other ST 55 strains in the pubMLST database have been isolated from clinical melioidosis in China and Malaysia. One ST 541 strain, MT 24.1, was isolated from soil. This ST was associated with a cluster of human deaths in Northern Vietnam and traced back to a contaminated borewell [15].

**Table 2:** Environmental and animal isolate details.

Environmental isolates	Date	Source	Province	MLST ST
SHT 1.2	25.04.2022	Soil	Hà Tĩnh	56
SHT 3.5	25.04.2022	Soil	Hà Tĩnh	41
SHT 4.5G	25.04.2022	Soil	Hà Tĩnh	307
SHT 5.1	25.04.2022	Soil	Hà Tĩnh	56
SHT 8.1	25.04.2022	Soil	Hà Tĩnh	55
SHT 9.1	25.04.2022	Soil	Hà Tĩnh	228
SHT 10.1	25.04.2022	Soil	Hà Tĩnh	381
MT 22.4	27.09.2016	Soil	Hà Tĩnh	2217*
MT 27.4.1	27.09.2016	Soil	Hà Tĩnh	2218*
MT 24.1	27.09.2016	Soil	Hà Tĩnh	541
**Animal isolates**				
VP74 G20.1	10.11.2020	Bear	Vinh Phuc	2219*
TY 01	5.11.2016	Pig	Nghe An	55

**Environmental and animal isolates sequenced in this study.** Ten environmental *B. pseudomallei* isolates were sequenced. Three from 2016 and seven from 2022. One soil isolate, SHT3.5 was ST 41. A bear from north of Hanoi was infected with a new ST and a pig from Nghe An was infected with ST 55.

**Fig 2:**
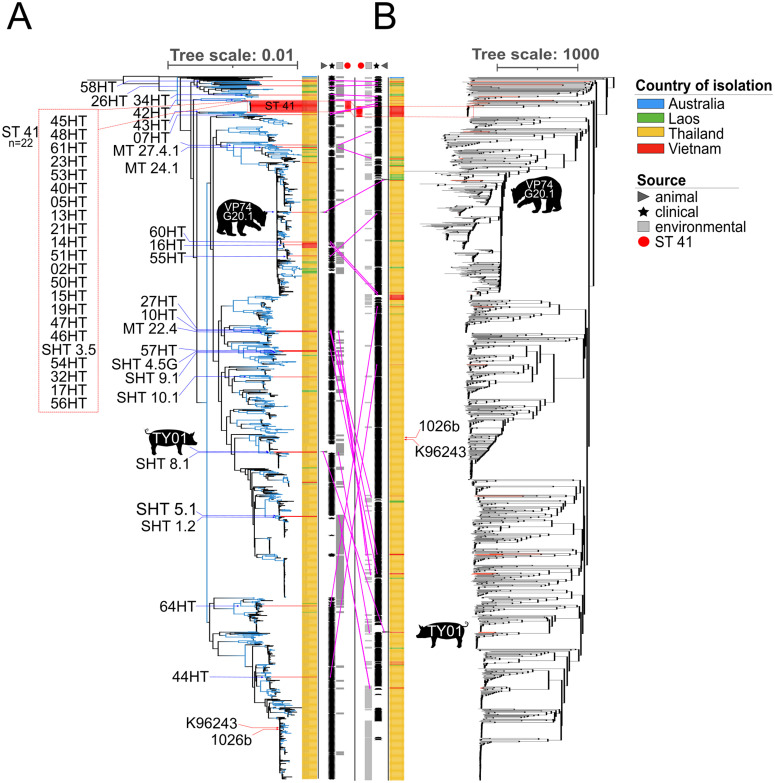
SNP and cgMLST phylogenetic tree comparison. Whole genome assemblies from 1468 *B. pseudomallei* strains including the 35 clinical, 10 environmental, and 2 animal isolates sequenced in this study (red branches) were analyzed by whole genome SNP analysis (A, left). High value (>90%) bootstrapped branches as determined using the ultrafast bootstrapping setting in IQ-TREE are colored blue. The PhAME analysis was compared to cgMLST analysis (B, right). The color strip indicates country of isolation. Australia, blue; Laos, green; Thailand, yellow; Vietnam, red. Between the two trees are columns indicating strain source including animal, clinical, or environmental. In the very center ST 41 strains are indicated by red dots and labeled in the dashed box. The locations of strains are labeled and indicated by blue arrows. The pink lines connecting A) and B) compare the location of the sequenced strains between the two trees. Tree scale in A) is in substitutions per site and in B) is cgMLST allele loci differences.

Whole genome SNP analysis of 1,468 *B. pseudomallei* strains from Australia and Southeast Asia, including the 47 from this work, by PhAME showed phylogenetic linkages of the isolates. For a comparison of typing methodologies, cgMLST analysis was performed on these strains using a previously published cgMLST scheme [11] and included isolates from Australia, Thailand, Laos, and Vietnam. SNP (Fig 2A) and cgMLST (Fig 2B) analysis revealed similar genetic relationships between the two methods. A majority of the *B. pseudomallei* sequenced in this work clustered towards the top end of the tree near Australian strains; considered the ancestral, more diverse *B. pseudomallei.* Local branching was conserved whether the tree was constructed by SNP or cgMLST analysis. Placement of more ancestral branches was different between SNP and cgMLST methods as indicated by shifts in tree location of strains in this work, with pink lines connecting strain leaves in trees made using the two different methods. Statistical bootstrapping was more supportive of strains placed closer together on the tree. The placement of some larger clades was not well supported, reflective of some of the differences in strain placement between SNP and cgMLST trees. The twenty-one ST 41 clinical isolates, were clustered closely to the ST 41 soil isolate SHT 3.5 (Fig 2A, red-dashed box). Both SNP and cgMLST identified two other clusters of clinical melioidosis strains and environmental isolates. One cluster includes soil isolate MT 22.4 and clinical isolates 10HT and 27HT. Another includes clinical isolate 57HT and the two soil isolates SHT 4.5G and SHT 9.1. The *B. pseudomallei* strain isolated from the bear, VP74 G20.1, was located near a cluster of *B. pseudomallei* from Vietnam as determined by wgSNP analysis. Other *B. pseudomallei* strains from our study that were phylogenetically related to bear isolate VP74 G20.1 were strains 16HT and 60HT isolated from human melioidosis. Core genome MLST placed this strain near several from Laos and somewhat further from the aforementioned human clinical isolates. The swine isolate TY01 from 2016 shared similar locations on both trees and, unsurprisingly, fell very close to soil isolate SHT 8.1 from 2022 since both were ST 55.

**Fig 3:**
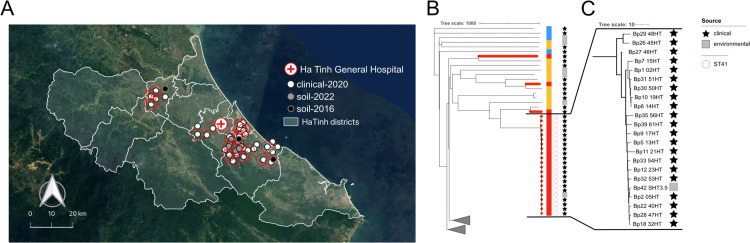
Detail of cgMLST typing done on ST 41 strains. **A)** A map of Ha Tinh province indicating the location of the clinical isolates from 2020 (white circles) and environmental isolates from 2016 and 2022 (black and gray points, respectively) recovered in relation to Ha Tinh Provincial General Hospital (red cross). The points indicated by red circles show which isolates were ST 41 strains. **B)** Whole genome SNP phylogenetics allowed further resolution of ST 41 strains recovered throughout the year across Ha Tinh province. Red branches indicate strains isolated and whole genome sequenced in this study. The color strip indicates country of origin. **C)** A zoomed in section of the SNP tree showing SNP differences in the core genomes of the ST 41 strains. Gray squares indicate during this study. One ST 41 soil isolate, SHT3.5, was recovered 2 years after the 2020 infection series. Province (https://purl.stanford.edu/mq929cm3505) and district (https://purl.stanford.edu/zq121fy4008) level base maps were used following the GADM license (https://gadm.org/license.html).

**Fig 4:**
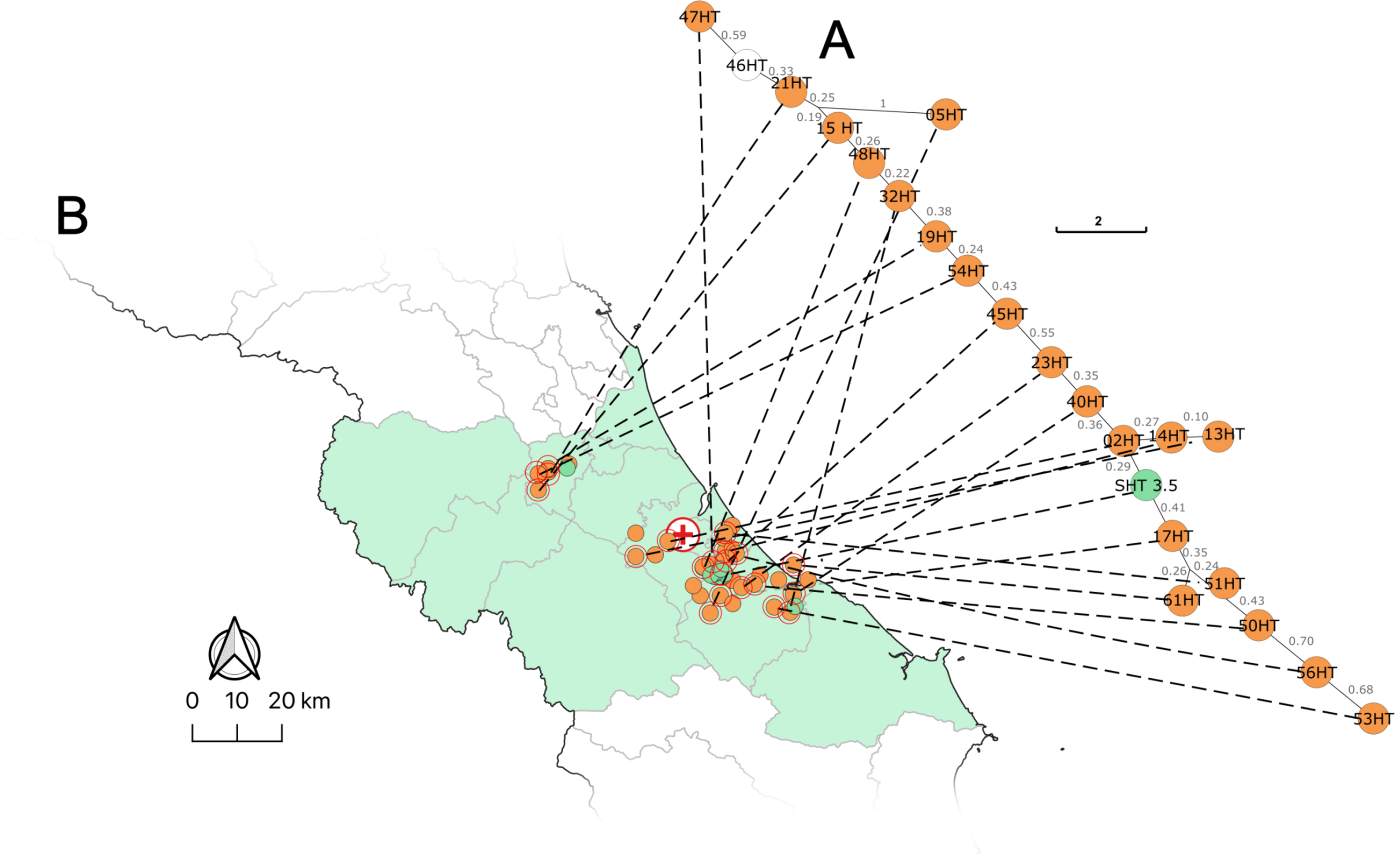
Phylogeographic comparison of cgMLST minimum spanning tree of ST 41 strains. **A)** A minimum spanning tree was created from the *B. pseudomallei* ST 41 cgMLST profiles. Strain names are located in the circles. The green filled circle is the only ST 41 soil isolate SHT 3.5 from 2022. The remainder are the twenty-one clinical ST 41 isolates. The unfilled circle is strain 46HT that lacked a *lipA* sequence but still fell in the ST 41 clade. The strains on the tree are connected to their location of isolation in Ha Tinh province by dashed lines. **B)** The map of Ha Tinh province (highlighted in green) showing the ST 41 strain isolation points circled in red. Country (https://purl.stanford.edu/dp747xv3917), province (https://purl.stanford.edu/mq929cm3505) and district (https://purl.stanford.edu/zq121fy4008) level base maps were used following the GADM license (https://gadm.org/license.html).

Twenty-one of the thirty-five clinical isolates (60%) identified in this work were ST 41. These ST 41 strains were isolated from patients residing at different locations in Ha Tinh province during the whole study year of 2020 (Fig 3A, red circles). The red circles on the map indicate their varied spatial locations in comparison to the location of Ha Tinh Provincial General Hospital. A closer look at ST 41 on the SNP tree revealed the highly clonal nature of the strains in the ST 41 cluster compared to other related clades (Fig 3B). In some cases, only a few SNPs in the core genomes separated the strains (Fig 3C). Strain SHT 3.5 was the ST 41 isolated from soil 2 years after the 2020 series of cases were diagnosed and was most closely related to strain 05HT from a melioidosis case in Cam Xuyen district, Ha Tinh province. The closely related strains, 40HT, 47HT, and 32HT, located on the distal end of the ST 41 SNP branch were also isolated from melioidosis cases in Cam Xuyen district. A minimum spanning tree (MST) produced using the cgMLST profiles (Fig 4A) of the ST 41 strains showed a phylogeographic correlation among genetically related strains and their geographic location of isolation from the southeast to the northwest of the study area (Fig 4B). There was not a clear temporal correlation between date of infection and strain location on the phylogenetic tree.

**Fig 5:**
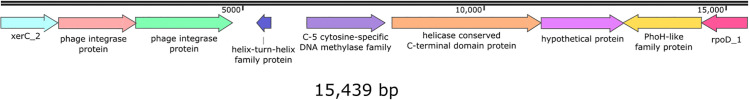
Schematic of genes present in *B. pseudomallei* ST 41 region 1 DNA. The 15,439 bp region of DNA found in ST 41 and at least one other *B. pseudomallei* strain (high virulence strain HBPUB10134a). Region 1 contains genes encoding in order, XerC tyrosine recombinase, two phage integrase proteins, helix-turn-helix DNA binding domain protein, a cytosine specific DNA methylase, helicase, hypothetical protein, a PhoH-like protein, and RNA polymerase sigma factor RpoD. This region is present in all ST 41 strains found in this work and others.

The finding that ST 41 *B. pseudomallei* were isolated from a majority of clinical cases in Ha Tinh province during the study period prompted a closer look at the genetic signatures that were unique to these strains. In case there were some virulence associated factors involved in what could be an ongoing epidemic of ST 41 melioidosis in the study region, a gene-by-gene BLAST was applied to all ST 41 strains in this work, other available ST 41 strains, and a selection of non-ST 41 *B. pseudomallei* to identify DNA regions found in ST 41 strains and a limited number of other *Burkholderiae* (Table 3). The largest region of ST 41-specific DNA (region 1) is ~ 15.4. kbp and encodes a PhoH-like and an RpoD sigma factor (Fig 5). Additional genes in this module encode a XerC tyrosine recombinase and two phage integrase proteins, suggesting this region may be the result of a lysogenic phage insertion. Both PhoH and RpoD-like proteins indicate the additional region could impact gene regulation and virulence. Nucleotide level homology results found a region of 99.95% DNA sequence identity across 100% of the module sequence in *B. pseudomallei* strain HBPUB10134a. This strain was isolated from a human infection in Thailand and has been associated with higher virulence in animal models [41,42]. *Burkholderia aenigmatica* strain CMCC(B)23010 had 86.40% nucleotide identity across 99% of the region while *Paraburkholderia hospita* strain mHSR1 had 81.63% nucleotide identity across 81.63% of the region. *Burkholderia aenigmatica* is a newly named species part of the *Burkholderia cepacia* complex [43] and *P. hospita* is a root associated bacteria that can induce resistance against bacterial plant pathogens [44,45]. Other alignments had coverage lower than 50%. *Burkholderia pseudomallei* strain HBPUB10134a is ST 228 and has some phylogenetic relationship with the ST 41 strains. Three other single gene regions present in ST 41 strains and no other *B. pseudomallei* encode hypothetical proteins. These regions were present in all ST 41 identified, even the Ha Tinh *B. pseudomallei* soil isolate SHT 3.5 from 2022, and could be developed as a molecular target to quickly differentiate ST41 and other strains that have this additional module.

**Table 3:** Diagnostic genes identified in Vietnam ST 41 strains.

Gene Region	Protein	blast functionality prediction
1	*xerC*_2	Tyrosine recombinase XerC
1	hypothetical protein	phage integrase family
1	hypothetical protein	phage integrase family
1	hypothetical protein	helix-turn-helix family protein
1	hypothetical protein	C-5 cytosine-specific DNA methylase family
1	hypothetical protein	helicase conserved C-terminal domain protein
1	hypothetical protein	hypothetical
1	hypothetical protein	phoH-like family protein
1	*rpoD*_1	RNA polymerase sigma factor RpoD
2	hypothetical protein	hypothetical
3	hypothetical protein	hypothetical
4	hypothetical protein	hypothetical

Genome blast analysis found a partial prophage was unique to Vietnam ST 41 strains. It was absent from other Vietnam isolates in this and other studies though found in high virulence type strain HBPUB10134a. Three additional genes encoding hypothetical proteins were also unique to ST 41. The region 1 genes can be developed as molecular targets to easily identify strains that have this module in the clinic or the environment.

## Discussion

Melioidosis is still not a reportable disease in Vietnam. Education and training have increased the knowledge and diagnosis of the disease in highly endemic regions. This study focused on sequencing and typing of strains at Ha Tinh Provincial General Hospital in Ha Tinh province located in North Central Vietnam following a concerted effort to increase awareness of human melioidosis. The blood of septicemic and pneumonic patients was the most frequent source material for isolating *B. pseudomallei.* Soil sampling efforts in the region from before and after the series of human cases provided a historical environmental isolate perspective, allowing contextual genetic snapshots of the human cases. Two animal isolates, one from a bear in Northern Vietnam and one from a pig in North Central Vietnam, provided a glimpse at strain types found in atypical and typical animal hosts.

Establishment of a sequencing pipeline in Vietnam was used to connect hospitals in endemic regions to academic and public health centers in Hanoi. Whole genome sequencing provided substantially more data than qPCR and allowed for detailed molecular epidemiological analysis traditional MLST cannot provide. SNP and sequence typing analysis, whether traditional seven marker MLST or several thousand marker cgMLST, can be extracted from the whole genome data and selectively applied depending on the goals. There was no direct correlation between STs in humans and those found in the environment. This is reflective of acquisition of bacteria from the environment, selection in the host, ST- specific pathogenic attributes and the genetic diversity of the pathogen in the soil niche. In this work, traditional MLST found that 60% of the clinical strains isolated from melioidosis patients at Ha Tinh Provincial General Hospital were a single ST, ST 41. The tendency for *B. pseudomallei* to have a large and expanding accessory genome by incorporating extraneous DNA by natural transformation and recombination can affect core genome-based phylogenetics (like wgSNP and cgMLST). The relatively large proportion of accessory genes limits the size of the core genome and inhibits accurate model determination during phylogenetic reconstruction [46]. This leads to uncertainty in phylogenetic branch placement like that observed between wgSNP and cgMLST trees. However, the high resolution of these techniques is still valuable where statistically supported. SNP and cgMLST analysis agreed with traditional MLST and the higher resolution analysis confirmed the clonal nature of the ST 41 strains. Some strains differed by only a few SNPs or cgMLST loci. The cgMLST minimum spanning tree comparison to location of the isolated strains showed a spatial correlation to the high-resolution genetic typing of the ST 41 strains. Even though enrichment of ST 41 strains was occurring in the patients, their genetics across Ha Tinh province could be generalized from the southwest to the northeast. The lack of infection foci was not surprising given the endemicity of *B. pseudomallei* in the soils of North Central Vietnam. There could be limited point-source infections within the case data but determining linkages without more epidemiological information is not possible.

Closer analysis of ST 41 strains found genetic regions that could explain the enrichment of this ST in the clinical series during 2020. Only one of the 10 soil isolates in this study was ST 41. Region 1 genes (Table 3) included phage integrase genes, supporting the hypothesis that a lysogenic phage transfer played a role in transfer of this gene module to ST 41 *B. pseudomallei* from a currently uncharacterized *B. pseudomallei* strain or possibly from near-neighbor like *Burkholderia cepacia*. Natural transformation is another mechanism by which naked DNA can be integrated into the genome of highly recombination proficient *B. pseudomallei* [47]*.* The predicted function of other genes in this genetic region supports a role for Region 1-encoded genes in human infections. PhoH-like proteins are responsible for upregulating virulence factors in response to phosphate starvation [48–50]. The human host is considered low in phosphate [49] and it is plausible that this additional PhoH protein could affect virulence mechanism induction. Adjacent to the *phoH* gene is an *rpoD* homologue. RpoD is a sigma factor (SigA or s70) that is known to activate housekeeping gene regulons important for exponential growth [51,52]. Another DNA binding protein in this region is a helix-turn-helix domain protein. With an abundance of gene regulatory elements present in this ~15 kbp region, Region 1 could be contributing to ST 41 infection rates in Ha Tinh and other regions including the US state of Georgia and is the subject of future investigations.

## Conclusions

This one-year provincial level cross-sectional study of human clinical cases at Ha Tinh Provincial General Hospital increases the molecular epidemiological data of melioidosis in an endemic region of Vietnam that has not been well studied. The 47 human, soil, and veterinary *B. pseudomallei* isolates sequenced as part of this work expands the whole genome data from strains in Vietnam. While the sample numbers in this study are small, interesting molecular epidemiological details were still obtained. A major *B. pseudomallei* human melioidosis causing sequence type was found during soil sampling. The animal samples currently have limited epidemiological value because only one from each species was identified, however, these data can provide a basis should future outbreaks among domesticated animals or wildlife occur. Limitations of this work include that exploratory soil sampling in southern, central, and northern Ha Tinh predated the clinical survey by several years, 2016 versus 2020, while follow-up sampling occurred in 2022. Despite asynchronous sampling, ST 55 was found in the swine from 2016 and in soil from 2022. Clinical ST41 from 2020 was found in soil from 2022. These examples reflect the long-term environmental stability of *B. pseudomallei*. Contemporaneous soil and clinical sampling could have generated a more correlated dataset. The current work provides a template for understanding the molecular epidemiology of melioidosis as diagnostic and training programs are moved further south of Ha Tinh into the South Central and Southern regions of Vietnam. Detailed molecular data of the *B. pseudomallei* in Vietnam is required as the foundation for place-specific diagnostic and vaccine development. Future work encompassing more dedicated soil, veterinary, and clinical studies are required to fully understand the role *B. pseudomallei* genetics are playing in human infections in Vietnam. This study represents an important step in moving *B. pseudomallei* towards a reportable disease in Vietnam. Partnering microbiology, clinical, veterinary, and public health representatives will be vital to understanding all facets of melioidosis in Vietnam.

## Supporting information

S1 Fig Map of all soil sampling sites in Ha Tinh. (TIFF)

S1 Table SRA and strain metadata for strains used to build the phylogenetic trees in Fig 2. (XLSX)

S2 Table Details of soil sampling sites coordinates, positivity rates, and year. (XLSX)
