# Supplementary figures and images for: Expanding the molecular epidemiology of melioidosis in North Central Vietnam
Source: PLoS Negl Trop Dis. 2026 Feb 9;20(2):e0013945. doi: 10.1371/journal.pntd.0013945 (PMC12900444; doi:10.1371/journal.pntd.0013945)

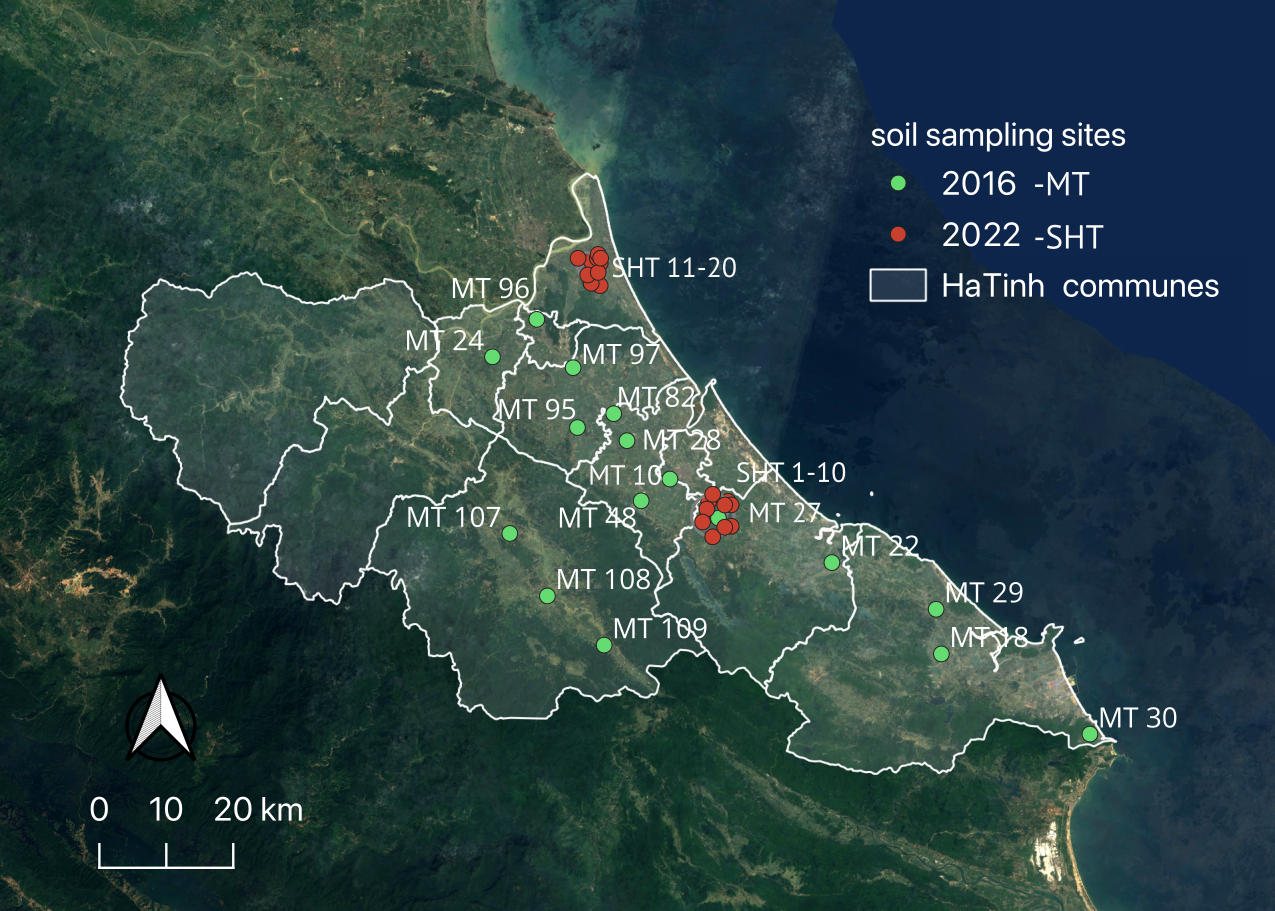

Supplement: S1 Fig — (TIFF) [file pntd.0013945.s001.tiff]
